# Ethyl 2-{[7-fluoro-4-oxo-3-(1*H*-1,2,4-triazol-1-yl)-4*H*-thio­chromen-2-yl]sulfan­yl}acetate

**DOI:** 10.1107/S1600536810027467

**Published:** 2010-07-21

**Authors:** Yang Li, Tao Xiao, Guang-yan Yu, Dong-liang Liu

**Affiliations:** a Department of Applied Chemistry, College of Science, Nanjing University of Technology, Nanjing 210009, People’s Republic of China

## Abstract

In the title compound, C_15_H_12_FN_3_O_3_S_2_, the two six-membered rings are essentially coplanar, their mean plnes making a dihedral angle of 1.1 (2)°. The carbonyl C, the two attached non-fused C atoms and the S atom deviate from the plane of the benzene ring by −0.046 (5), −0.017 (5), 0.000 (6), 0.026 (4) Å, respectively. The angle between the mean planes of the triazole ring and the sulfur heterocycle is 53.3 (1)°. In the crystal, inter­molecular C—H⋯O hydrogen bonds link the mol­ecules in a stacked arrangement along the *a* axis.

## Related literature

For related compounds containing a 4*H*-thio­chromen-4-one fragment, see: Adams *et al.* (1991 ▶); Nakazumi *et al.* (1992 ▶); Weiss *et al.* (2008 ▶); Li *et al.* (2010 ▶). For bond-length data, see: Allen *et al.* (1987 ▶).
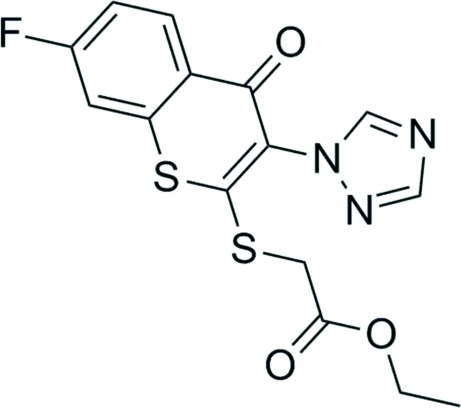


## Experimental

### 

#### Crystal data

C_15_H_12_FN_3_O_3_S_2_
*M* *_r_* = 365.40
Monoclinic, 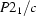

*a* = 9.3890 (19) Å
*b* = 8.2430 (16) Å
*c* = 20.861 (4) Å
β = 100.72 (3)°
*V* = 1586.3 (5) Å^3^
*Z* = 4
Mo *K*α radiation
μ = 0.37 mm^−1^
*T* = 293 K
0.30 × 0.20 × 0.10 mm

#### Data collection

Enraf–Nonius CAD-4 diffractometer
Absorption correction: ψ scan (North *et al.*, 1968 ▶) *T*_min_ = 0.898, *T*_max_ = 0.964
3053 measured reflections
2867 independent reflections
2186 reflections with *I* > 2σ(*I*)
*R*_int_ = 0.013
3 standard reflections every 200 reflections  intensity decay: 1%

#### Refinement

*R*[*F* ^2^ > 2σ(*F* ^2^)] = 0.044
*wR*(*F* ^2^) = 0.151
*S* = 1.00
2867 reflections
217 parameters
H-atom parameters constrained
Δρ_max_ = 0.22 e Å^−3^
Δρ_min_ = −0.32 e Å^−3^

### 

Data collection: *CAD-4 EXPRESS* (Enraf–Nonius, 1985 ▶); cell refinement: *CAD-4 EXPRESS*; data reduction: *XCAD4* (Harms & Wocadlo, 1995 ▶); program(s) used to solve structure: *SHELXS97* (Sheldrick, 2008 ▶); program(s) used to refine structure: *SHELXL97* (Sheldrick, 2008 ▶); molecular graphics: *PLATON* (Spek, 2009 ▶); software used to prepare material for publication: *SHELXTL* (Sheldrick, 2008 ▶).

## Supplementary Material

Crystal structure: contains datablocks I, global. DOI: 10.1107/S1600536810027467/zq2047sup1.cif

Structure factors: contains datablocks I. DOI: 10.1107/S1600536810027467/zq2047Isup2.hkl

Additional supplementary materials:  crystallographic information; 3D view; checkCIF report

## Figures and Tables

**Table 1:** Hydrogen-bond geometry (Å, °)

*D*—H⋯*A*	*D*—H	H⋯*A*	*D*⋯*A*	*D*—H⋯*A*
C4—H4*A*⋯O2^i^	0.97	2.47	3.199 (4)	131
C11—H11*A*⋯O2^ii^	0.93	2.43	3.276 (4)	151

Symmetry codes: (i) 
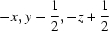
; (ii) 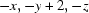.

**Fig. 1.**
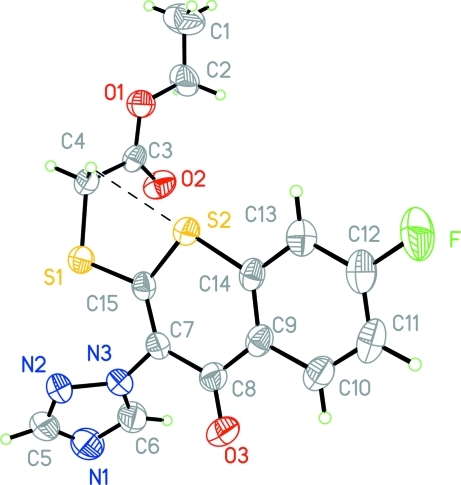
The molecular structure of (I), with the atom-numbering scheme. Displacement ellipsoids are drawn at the 50% probability level.

**Fig. 2.**
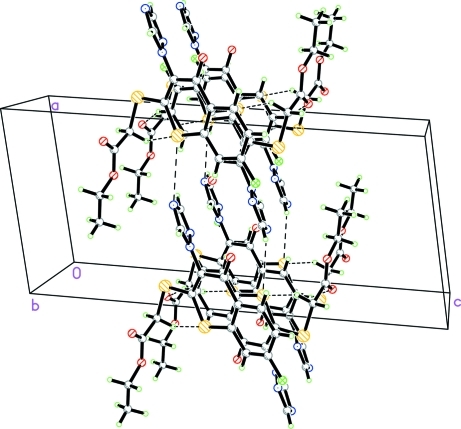
A packing diagram of (I). Intra- and inter-molecular interactions are shown as dashed lines.

## supplementary crystallographic information

### Comment

The title compound, ethyl 2-((7-fluoro-4-oxo-3-(1*H*-1,2,4-triazol-1-yl)-4*H*-thiochromen-2-yl)thio) acetate (I), is a new molecule which has a potential use as antifungal. We herein report its crystal structure.

The molecular structure of (I) is shown in Fig. 1, and selected geometric parameters are given in Table 1. The bond lengths and angles (Table 1) are within normal ranges (Allen *et al.*, 1987). The two-ring system is essentially planar [angle between the mean planes = 1.1 (2)°]. The atoms C7, C8, C15 and S2 deviate from the benzene ring by -0.046 (5), -0.017 (5), 0.000 (6), 0.026 (4) Å, respectively. The angle between the mean planes of the triazole ring and the sulfur heterocycle is 53.3 (1)°.

In the crystal packing, a weak intramolecular C4—H4B···S2 interaction is observed, and intermolecular C—H···O hydrogen bonds link the molecules in a stacked arrangement along the *a* axis.

### Experimental

CS_2_ (2.0 g, 26.3 mmol) was dropwise added to a solution of 1-(2,4- difluorophenyl)-2-(1*H*-1,2,4-triazol-1-yl)ethanone (5 g, 22.4 mmol) in DMSO (20 ml) containing NaOH (1.8 g, 45 mmol). The yellow solution was stirred for about 2 h at room temperature. Then ethyl bromoacetate (3.8 g, 22.4 mmol) was dropwise added to the intermediate. After 3 h, the solution was poured into water (50 ml). The crystalline product was isolated by filtration, and washed with water (300 ml). The crystals were obtained by dissolving (I) in acetone (20 ml) and evaporating acetone slowly at room temperature for about 7 d.

### Refinement

H atoms were positioned geometrically with C—H = 0.93 Å for aromatic H atoms, C—H = 0.97 Å for methylene H atoms,and with C—H = 0.96 Å for methyl H atoms, and constrained to ride on their parent atoms with *U*_iso_(H) = *xU*_eq_(C), where *x* = 1.2 for aromatic and methylene H atoms and *x* = 1.5 for methyl H atoms.

### Crystal data

**Table:**

C_15_H_12_FN_3_O_3_S_2_	*F*(000) = 752
*M**_r_* = 365.40	*D*_x_ = 1.530 Mg m^−^^3^
Monoclinic, *P*2_1_/*c*	Melting point: 397 K
Hall symbol: -P 2ybc	Mo *K*α radiation, λ = 0.71073 Å
*a* = 9.3890 (19) Å	Cell parameters from 25 reflections
*b* = 8.2430 (16) Å	θ = 9–14°
*c* = 20.861 (4) Å	µ = 0.37 mm^−^^1^
β = 100.72 (3)°	*T* = 293 K
*V* = 1586.3 (5) Å^3^	Block, pink
*Z* = 4	0.30 × 0.20 × 0.10 mm

### Data collection

**Table:**

Enraf–Nonius CAD-4 diffractometer	2186 reflections with *I* > 2σ(*I*)
Radiation source: fine-focus sealed tube	*R*_int_ = 0.013
graphite	θ_max_ = 25.3°, θ_min_ = 2.0°
ω/2θ scans	*h* = 0→11
Absorption correction: ψ scan (North *et al.*, 1968)	*k* = 0→9
*T*_min_ = 0.898, *T*_max_ = 0.964	*l* = −25→24
3053 measured reflections	3 standard reflections every 200 reflections
2867 independent reflections	intensity decay: 1%

### Refinement

**Table:**

Refinement on *F*^2^	Primary atom site location: structure-invariant direct methods
Least-squares matrix: full	Secondary atom site location: difference Fourier map
*R*[*F*^2^ > 2σ(*F*^2^)] = 0.044	Hydrogen site location: inferred from neighbouring sites
*wR*(*F*^2^) = 0.151	H-atom parameters constrained
*S* = 1.00	*w* = 1/[σ^2^(*F*_o_^2^) + (0.1*P*)^2^ + 0.170*P*] where *P* = (*F*_o_^2^ + 2*F*_c_^2^)/3
2867 reflections	(Δ/σ)_max_ < 0.001
217 parameters	Δρ_max_ = 0.22 e Å^−^^3^
0 restraints	Δρ_min_ = −0.32 e Å^−^^3^

### Special details

**Table:**

Geometry. All e.s.d.'s (except the e.s.d. in the dihedral angle between two l.s. planes) are estimated using the full covariance matrix. The cell e.s.d.'s are taken into account individually in the estimation of e.s.d.'s in distances, angles and torsion angles; correlations between e.s.d.'s in cell parameters are only used when they are defined by crystal symmetry. An approximate (isotropic) treatment of cell e.s.d.'s is used for estimating e.s.d.'s involving l.s. planes.
Refinement. Refinement of *F*^2^ against ALL reflections. The weighted *R*-factor *wR* and goodness of fit *S* are based on *F*^2^, conventional *R*-factors *R* are based on *F*, with *F* set to zero for negative *F*^2^. The threshold expression of *F*^2^ > σ(*F*^2^) is used only for calculating *R*-factors(gt) *etc*. and is not relevant to the choice of reflections for refinement. *R*-factors based on *F*^2^ are statistically about twice as large as those based on *F*, and *R*- factors based on ALL data will be even larger.

### Fractional atomic coordinates and isotropic or equivalent isotropic displacement parameters (Å^2^)

**Table:**

	*x*	*y*	*z*	*U*_iso_*/*U*_eq_	
F	0.2497 (3)	0.9099 (3)	−0.12075 (10)	0.0728 (7)	
S1	−0.10898 (8)	0.52611 (10)	0.15706 (4)	0.0434 (3)	
O1	0.3016 (2)	0.6102 (3)	0.23375 (11)	0.0473 (6)	
N1	−0.5765 (3)	0.6974 (5)	0.09253 (17)	0.0721 (10)	
C1	0.5439 (4)	0.6893 (6)	0.2766 (2)	0.0871 (15)	
H1B	0.6065	0.7781	0.2928	0.131*	
H1C	0.5738	0.6438	0.2388	0.131*	
H1D	0.5494	0.6077	0.3098	0.131*	
S2	0.06614 (8)	0.65095 (9)	0.06388 (3)	0.0375 (2)	
O2	0.1081 (3)	0.7704 (3)	0.22875 (11)	0.0554 (6)	
N2	−0.4048 (3)	0.5142 (4)	0.07917 (15)	0.0562 (8)	
C2	0.3947 (4)	0.7475 (5)	0.2588 (2)	0.0614 (10)	
H2B	0.3884	0.8312	0.2257	0.074*	
H2C	0.3635	0.7935	0.2967	0.074*	
N3	−0.3597 (3)	0.6709 (3)	0.07017 (13)	0.0472 (7)	
O3	−0.3528 (3)	0.8531 (3)	−0.03655 (13)	0.0646 (7)	
C3	0.1619 (3)	0.6420 (4)	0.21998 (13)	0.0372 (7)	
C4	0.0790 (3)	0.4931 (3)	0.19245 (14)	0.0382 (7)	
H4A	0.0840	0.4136	0.2271	0.046*	
H4B	0.1269	0.4468	0.1593	0.046*	
C5	−0.5331 (4)	0.5401 (6)	0.0927 (2)	0.0673 (11)	
H5A	−0.5917	0.4556	0.1020	0.081*	
C6	−0.4652 (4)	0.7752 (5)	0.07764 (19)	0.0601 (10)	
H6A	−0.4601	0.8871	0.0729	0.072*	
C7	−0.2261 (3)	0.6990 (4)	0.04953 (14)	0.0378 (7)	
C8	−0.2363 (3)	0.7960 (4)	−0.00981 (15)	0.0428 (7)	
C9	−0.1040 (3)	0.8222 (3)	−0.03656 (14)	0.0386 (7)	
C10	−0.1155 (4)	0.9141 (4)	−0.09443 (15)	0.0473 (8)	
H10A	−0.2052	0.9558	−0.1138	0.057*	
C11	0.0020 (4)	0.9427 (4)	−0.12239 (16)	0.0521 (9)	
H11A	−0.0068	1.0032	−0.1606	0.063*	
C12	0.1336 (4)	0.8808 (4)	−0.09318 (16)	0.0490 (8)	
C13	0.1538 (4)	0.7913 (4)	−0.03709 (15)	0.0432 (7)	
H13A	0.2445	0.7502	−0.0188	0.052*	
C14	0.0329 (3)	0.7637 (3)	−0.00815 (13)	0.0357 (7)	
C15	−0.1023 (3)	0.6355 (3)	0.08501 (14)	0.0362 (7)	

### Atomic displacement parameters (Å^2^)

**Table:**

	*U*^11^	*U*^22^	*U*^33^	*U*^12^	*U*^13^	*U*^23^
F	0.0870 (16)	0.0758 (15)	0.0678 (14)	−0.0063 (13)	0.0462 (12)	0.0109 (12)
S1	0.0411 (4)	0.0490 (5)	0.0400 (4)	−0.0036 (4)	0.0071 (3)	0.0107 (3)
O1	0.0433 (12)	0.0353 (12)	0.0611 (14)	−0.0009 (10)	0.0037 (10)	−0.0065 (10)
N1	0.0419 (17)	0.088 (3)	0.088 (2)	−0.0022 (17)	0.0173 (16)	0.019 (2)
C1	0.052 (2)	0.074 (3)	0.128 (4)	−0.007 (2)	−0.003 (2)	−0.028 (3)
S2	0.0376 (4)	0.0383 (4)	0.0366 (4)	0.0003 (3)	0.0071 (3)	0.0057 (3)
O2	0.0633 (15)	0.0396 (13)	0.0589 (15)	0.0130 (11)	−0.0001 (11)	−0.0129 (11)
N2	0.0419 (16)	0.0521 (18)	0.072 (2)	−0.0130 (13)	0.0024 (14)	0.0184 (15)
C2	0.055 (2)	0.045 (2)	0.083 (3)	−0.0150 (17)	0.0077 (19)	−0.0131 (18)
N3	0.0371 (14)	0.0473 (16)	0.0551 (16)	−0.0067 (12)	0.0029 (12)	0.0093 (13)
O3	0.0485 (14)	0.0726 (18)	0.0679 (16)	0.0057 (13)	−0.0015 (12)	0.0296 (14)
C3	0.0488 (18)	0.0330 (16)	0.0290 (14)	0.0022 (14)	0.0051 (12)	0.0027 (12)
C4	0.0441 (16)	0.0322 (16)	0.0368 (15)	0.0017 (13)	0.0038 (13)	0.0026 (13)
C5	0.044 (2)	0.078 (3)	0.076 (3)	−0.017 (2)	0.0012 (18)	0.025 (2)
C6	0.0440 (19)	0.058 (2)	0.081 (3)	0.0040 (18)	0.0176 (18)	0.013 (2)
C7	0.0348 (15)	0.0327 (16)	0.0456 (16)	−0.0044 (13)	0.0067 (12)	0.0033 (13)
C8	0.0470 (18)	0.0354 (16)	0.0426 (16)	−0.0047 (14)	−0.0004 (14)	0.0027 (14)
C9	0.0526 (18)	0.0261 (15)	0.0355 (15)	−0.0016 (13)	0.0038 (13)	−0.0009 (12)
C10	0.063 (2)	0.0363 (17)	0.0384 (16)	−0.0023 (16)	−0.0007 (15)	0.0038 (14)
C11	0.084 (3)	0.0357 (18)	0.0392 (17)	−0.0022 (18)	0.0171 (17)	0.0065 (14)
C12	0.069 (2)	0.0382 (18)	0.0451 (18)	−0.0060 (16)	0.0244 (16)	−0.0024 (15)
C13	0.0524 (18)	0.0377 (17)	0.0427 (16)	−0.0012 (15)	0.0168 (14)	−0.0017 (14)
C14	0.0496 (18)	0.0242 (14)	0.0333 (15)	−0.0043 (13)	0.0074 (13)	−0.0022 (12)
C15	0.0419 (16)	0.0294 (15)	0.0373 (15)	−0.0054 (13)	0.0077 (12)	−0.0009 (12)

### Geometric parameters (Å, °)

**Table:**

F—C12	1.345 (4)	N3—C7	1.419 (4)
S1—C15	1.764 (3)	O3—C8	1.226 (4)
S1—C4	1.802 (3)	C3—C4	1.509 (4)
O1—C3	1.316 (4)	C4—H4A	0.9700
O1—C2	1.466 (4)	C4—H4B	0.9700
N1—C6	1.311 (4)	C5—H5A	0.9300
N1—C5	1.359 (5)	C6—H6A	0.9300
C1—C2	1.462 (5)	C7—C15	1.361 (4)
C1—H1B	0.9600	C7—C8	1.461 (4)
C1—H1C	0.9600	C8—C9	1.469 (4)
C1—H1D	0.9600	C9—C14	1.397 (4)
S2—C15	1.723 (3)	C9—C10	1.412 (4)
S2—C14	1.745 (3)	C10—C11	1.361 (5)
O2—C3	1.201 (4)	C10—H10A	0.9300
N2—C5	1.305 (5)	C11—C12	1.370 (5)
N2—N3	1.383 (4)	C11—H11A	0.9300
C2—H2B	0.9700	C12—C13	1.366 (5)
C2—H2C	0.9700	C13—C14	1.400 (4)
N3—C6	1.342 (4)	C13—H13A	0.9300
			
C15—S1—C4	103.82 (14)	N1—C5—H5A	121.8
C3—O1—C2	115.3 (3)	N1—C6—N3	110.6 (4)
C6—N1—C5	102.4 (3)	N1—C6—H6A	124.7
C2—C1—H1B	109.5	N3—C6—H6A	124.7
C2—C1—H1C	109.5	C15—C7—N3	119.1 (3)
H1B—C1—H1C	109.5	C15—C7—C8	125.8 (3)
C2—C1—H1D	109.5	N3—C7—C8	115.1 (3)
H1B—C1—H1D	109.5	O3—C8—C7	120.5 (3)
H1C—C1—H1D	109.5	O3—C8—C9	121.1 (3)
C15—S2—C14	103.51 (15)	C7—C8—C9	118.4 (3)
C5—N2—N3	101.2 (3)	C14—C9—C10	117.7 (3)
C1—C2—O1	108.4 (3)	C14—C9—C8	124.3 (3)
C1—C2—H2B	110.0	C10—C9—C8	117.9 (3)
O1—C2—H2B	110.0	C11—C10—C9	121.4 (3)
C1—C2—H2C	110.0	C11—C10—H10A	119.3
O1—C2—H2C	110.0	C9—C10—H10A	119.3
H2B—C2—H2C	108.4	C10—C11—C12	118.7 (3)
C6—N3—N2	109.3 (3)	C10—C11—H11A	120.7
C6—N3—C7	130.1 (3)	C12—C11—H11A	120.7
N2—N3—C7	120.3 (3)	F—C12—C13	117.9 (3)
O2—C3—O1	124.9 (3)	F—C12—C11	118.6 (3)
O2—C3—C4	125.0 (3)	C13—C12—C11	123.5 (3)
O1—C3—C4	110.1 (2)	C12—C13—C14	117.6 (3)
C3—C4—S1	115.4 (2)	C12—C13—H13A	121.2
C3—C4—H4A	108.4	C14—C13—H13A	121.2
S1—C4—H4A	108.4	C9—C14—C13	121.1 (3)
C3—C4—H4B	108.4	C9—C14—S2	123.5 (2)
S1—C4—H4B	108.4	C13—C14—S2	115.4 (2)
H4A—C4—H4B	107.5	C7—C15—S2	124.3 (2)
N2—C5—N1	116.4 (3)	C7—C15—S1	119.8 (2)
N2—C5—H5A	121.8	S2—C15—S1	115.82 (17)
			
C3—O1—C2—C1	175.4 (3)	C7—C8—C9—C10	179.1 (3)
C5—N2—N3—C6	−1.3 (4)	C14—C9—C10—C11	1.1 (4)
C5—N2—N3—C7	−175.4 (3)	C8—C9—C10—C11	−179.6 (3)
C2—O1—C3—O2	−2.9 (5)	C9—C10—C11—C12	−0.2 (5)
C2—O1—C3—C4	177.9 (3)	C10—C11—C12—F	−179.7 (3)
O2—C3—C4—S1	11.9 (4)	C10—C11—C12—C13	−0.1 (5)
O1—C3—C4—S1	−168.9 (2)	F—C12—C13—C14	179.0 (3)
C15—S1—C4—C3	70.5 (2)	C11—C12—C13—C14	−0.6 (5)
N3—N2—C5—N1	0.9 (5)	C10—C9—C14—C13	−1.8 (4)
C6—N1—C5—N2	−0.1 (5)	C8—C9—C14—C13	178.9 (3)
C5—N1—C6—N3	−0.8 (4)	C10—C9—C14—S2	179.0 (2)
N2—N3—C6—N1	1.4 (4)	C8—C9—C14—S2	−0.2 (4)
C7—N3—C6—N1	174.8 (3)	C12—C13—C14—C9	1.6 (4)
C6—N3—C7—C15	131.6 (4)	C12—C13—C14—S2	−179.2 (2)
N2—N3—C7—C15	−55.7 (4)	C15—S2—C14—C9	0.6 (3)
C6—N3—C7—C8	−48.6 (5)	C15—S2—C14—C13	−178.6 (2)
N2—N3—C7—C8	124.1 (3)	N3—C7—C15—S2	176.7 (2)
C15—C7—C8—O3	−177.0 (3)	C8—C7—C15—S2	−3.0 (5)
N3—C7—C8—O3	3.3 (4)	N3—C7—C15—S1	−2.2 (4)
C15—C7—C8—C9	3.4 (5)	C8—C7—C15—S1	178.1 (2)
N3—C7—C8—C9	−176.4 (3)	C14—S2—C15—C7	0.9 (3)
O3—C8—C9—C14	178.7 (3)	C14—S2—C15—S1	179.85 (16)
C7—C8—C9—C14	−1.6 (4)	C4—S1—C15—C7	−171.9 (2)
O3—C8—C9—C10	−0.6 (5)	C4—S1—C15—S2	9.1 (2)

### Hydrogen-bond geometry (Å, °)

**Table:**

*D*—H···*A*	*D*—H	H···*A*	*D*···*A*	*D*—H···*A*
C4—H4A···O2^i^	0.97	2.47	3.199 (4)	131
C4—H4B···S2	0.97	2.59	2.963 (3)	103
C11—H11A···O2^ii^	0.93	2.43	3.276 (4)	151

Symmetry codes: (i) −*x*, *y*−1/2, −*z*+1/2; (ii) −*x*, −*y*+2, −*z*.
